# Simultaneous construction strategy using two types of fluorescent markers for HVT vector vaccine against infectious bursal disease and H9N2 avian influenza virus by NHEJ-CRISPR/Cas9

**DOI:** 10.3389/fvets.2024.1385958

**Published:** 2024-05-13

**Authors:** Jun-Feng Zhang, Ke Shang, Sang-Won Kim, Jong-Yeol Park, Bai Wei, Hyung-Kwan Jang, Min Kang, Se-Yeoun Cha

**Affiliations:** ^1^College of Medical Technology and Engineering, Henan University of Science and Technology, Luoyang, China; ^2^Department of Avian Diseases, College of Veterinary Medicine and Center for Avian Disease, Jeonbuk National University, Iksan, Republic of Korea; ^3^College of Animal Science and Technology, Luoyang Key Laboratory of Live Carrier Biomaterial and Animal Disease Prevention and Control, Henan University of Science and Technology, Luoyang, China; ^4^Bio Disease Control (BIOD) Co., Ltd., Iksan, Republic of Korea

**Keywords:** Turkey herpesvirus, CRISPR/Cas9, NHEJ, infectious bursal disease virus, G2d

## Abstract

Recently, herpesvirus of turkeys (HVT), which was initially employed as a vaccine against Marek’s disease (MD), has been shown to be a highly effective viral vector for producing recombinant vaccines that can simultaneously express the protective antigens of multiple poultry diseases. Prior to the development of commercial HVT-vectored dual-insert vaccines, the majority of HVT-vectored vaccines in use only contained a single foreign gene and were often generated using time-consuming and inefficient traditional recombination methods. The development of multivalent HVT-vectored vaccines that induce simultaneous protection against several avian diseases is of great value. In particular, efficacy interference between individual recombinant HVT vaccines can be avoided. Herein, we demonstrated the use of CRISPR/Cas9 gene editing technology for the insertion of an IBDV (G2d) VP2 expression cassette into the UL45/46 region of the recombinant rHVT-HA viral genome to generate the dual insert rHVT-VP2-HA recombinant vaccine. The efficacy of this recombinant virus was also evaluated in specific pathogen-free (SPF) chickens. PCR and sequencing results showed that the recombinant virus rHVT-VP2-HA was successfully constructed. Vaccination with rHVT-VP2-HA produced high levels of specific antibodies against IBDV (G2d) and H9N2/Y280. rHVT-VP2-HA can provide 100% protection against challenges with IBDV (G2d) and H9N2/Y280. These results demonstrate that rHVT-VP2-HA is a safe and highly efficacious vaccine for the simultaneous control of IBDV (G2d) and H9N2/Y280.

## 1 Introduction

Acute, highly contagious, immunosuppressive infectious bursal disease (IBD), which often referred to as Gumboro disease, affects young chickens and is caused by the IBD virus (IBDV), a member of the Birnaviridae family, genus Avibirnavirus. IBDV targets and destroys the precursors of antibody-producing B cells in the bursa of Fabricius of young chickens, thus inducing immunosuppression, which leads to vaccination failures and an increased susceptibility to other infectious agents (1). IBDV has two known serotypes (I and II); however, only serotype I is pathogenic to chickens. According to pathogenicity and antigenicity, serotype I is divided into four primary pathotypes: classical virulent (cv), antigenic variation (av), very virulent (vv), and attenuated (at). However, a new classification of IBDVs with seven genogroups (G1–G7) has been proposed due to the rapid occurrence of genetic variations in the hypervariable (hv) VP2 area. The cv/atIBDV, avIBDV, and vvIBV strains correspond to G1, G2 and G3, respectively (2, 3). G2 has been further geographically divided into the sub-lineages G2a, G2b, G2c, and G2d. Recently, studies have shown that IBDV, with G2d as the dominant genogroup, is highly prevalent in South Korea and poses a new threat to poultry farms (4). Studies have shown that the current vaccines cannot provide efficient protection against this novel variant. The lack of an effective antigen-matched vaccine has led to an increasing prevalence of this new genotype on poultry farms. The development of a matched vaccine against G2d variant is urgently needed.

In 1966, the low-pathogenic avian influenza virus (LPAIV) H9N2 AIV was initially discovered in flocks of turkeys in Wisconsin, USA (5). Since then, H9N2 AIVs have been found in mammals, domestic poultry, and wild birds all across the world (6–8). H9N2 AIV has become the most common influenza virus subtype in poultry, causing significant economic losses and a threat to public health. H9N2 AIVs can be roughly divided into Eurasian and American lineages (9, 10). The G1-like lineage (represented by A/quail/Hong Kong/G1/1997; H9N2/G1), the Y280-like lineage (represented by A/duck/Hong Kong/Y280/1997; H9N2/Y280), and the Y439-like lineage (represented by A/duck/Hong Kong/Y439/1997; H9N2/Y439) are the three sub-lineages that compose the Eurasian lineage H9N2 AIVs (11). The first H9N2 AIV outbreak in Korea occurred in 1996. Since then, H9N2/Y439 has been prevalent and continuously evolved through reassortment in live bird markets (12). Since 2007, the Korean government has permitted the use of an inactivated H9N2/Y439 vaccine (Based on A/chicken/Korea/01310/2001 strain). Therefore, outbreaks of H9N2 became less common in broilers and layers (13). However, due to the continued spread of H9N2 AIV in Korean native chickens (KNCs) from live bird market (LBM), the Y439-like lineage evolved through reassortment with Eurasian aquatic bird-derived LPAIV, leading to the emergence of novel H9N2/Y439 with altered pathogenicity (14, 15). In June 2020, a novel H9N2 AIV belonging to the Y280-like lineage was identified in KNCs during national surveillance (16). Studies have shown that the current commercial vaccines cannot provide complete protection against the H9N2/Y280 (17). Therefore, the development of a Y280-like lineage-matched vaccine is urgently needed.

Herpesvirus of turkeys (HVT), also known as Mardivirus meleagridalpha 1, has been used since the 1970s in vaccines against Marek’s disease (MD). It is a member of the family Herpesviridae, subfamily Alphaherpesvirinae, and genus Mardivirus (18). HVT is frequently utilized as a vaccine vector for the expression of heterologous antigens against a number of avian diseases (19–23). HVT has several distinct benefits as a vaccine vector. It is non-pathogenic to chickens and HVT cannot spread horizontally may prove to low potential risks for released HVT vector or genetically engineered HVT vector vaccine in environment in long-term impacts; therefore, it is very safe to use (24). The large genome of HVT contains many non-essential regions; thus, multiple foreign genes can be inserted simultaneously, making the development of multivalent vaccines possible. HVT is a cell-associated virus and can induce lifelong immunity (25).

Various commercially available HVT-vectored vaccines are currently used to protect chicken flocks against several major diseases such as avian influenza virus (AIV), IBDV, infectious laryngotracheitis virus (ILTV), and Newcastle disease virus (NDV) (26–30). The widespread commercial use of these recombinant vaccines has demonstrated their critical role in protecting flocks and addressing issues with live vaccines such as vaccination responses in the respiratory tract and potential pathogenicity reversal. However, these vaccines are monovalent. They each contain only one protective antigen against one pathogen; thus, chickens can only be protected from one disease at a time. It is not possible to combine different monovalent HVT-vectored vaccines in one vaccination because this would lead to interference. Reports have indicated that applying two vectored vaccines using the same HVT vector reduces the efficacy of one or both vaccines (31).

The clustered regularly interspaced short palindromic repeats (CRISPR)/CRISPR-associated 9 (Cas9) system has recently acquired prominence because of its versatility and specificity. In this system, a 20-nucleotide target sequence adjacent to a 5’-NGG-3’ protospacer adjacent motif (PAM) is recognized by a single-guide RNA (sgRNA), and Cas9 initiates a double-strand break (DSB) in this target sequence. DSBs can subsequently be repaired by either the error-prone non-homologous end-joining (NHEJ) or high-fidelity homology-directed repair (HDR) pathway (32). The NHEJ-CRISPR/Cas9 system has higher insertion efficiency than the HDR-CRISPR/Cas9 system (33). The NHEJ-CRISPR/Cas9 system has revolutionized genome editing and enables rapid and precise gene modification.

In this study, we utilized NHEJ-CRISPR/Cas9 technology to express the VP2 protein of IBDV (G2d) and the HA protein of H9N2/Y280 to simultaneously provide protection against IBDV and H9N2 using a single vaccine. As a result, a double insertion recombinant virus named rHVT-VP2-HA was generated. The efficacy of this recombinant virus was assessed for its protective function against challenges by IBDV (G2d) and H9N2/Y280 in specific pathogen-free (SPF) chickens.

## 2 Materials and methods

### 2.1 Viruses and cell culture

The HVT Fc126 strain (GenBank accession number, AF291866.1) was stored in liquid nitrogen, and the IBDV (G2d) strain A21-ETC-014 and H9N2/Y280 strain A21-MRA-003 were identified and preserved at −70°C in our laboratory (34, 35). Primary chicken embryo fibroblasts (CEFs) from 10-day old specific pathogen-free (SPF) chicken embryos (Sunrise Farms, Inc., Stuarts Draft, VA, USA) were prepared according to previously described (36). All HVT strains were propagated in primary or secondary CEFs. Isolation and titration of IBDV (G2d) performed in 10-day old SPF chicken embryonated eggs via the chorioallantoic membrane (CAM) route, and H9N2/Y280 virus was performed in 9-day old SPF chicken embryonated eggs via the allantoic cavity (AC) route as previously described (37, 38). For the titration of IBDV (G2d) and H9N2/Y280, the 50% endpoints were calculated using the Reed and Muench’s method for EID_50_ (39).

### 2.2 Construction of CAS9/GRNA expression plasmids and donor plasmids

The gRNA targeting the UL45/46 region of the HVT genome was designed using the CRISPR guide RNA (gRNA) design online tool CHOPCHOP and cloned into the CRISPR expression plasmid pSpCas9(BB)-2A-Puro (PX459) V2.0 (Addgene, Watertown, MA, USA) to yield PX459-UL45/46-gRNA by inserting synthesized primers UL45/46-gRNA-F/R into BbsI restriction sites (40). Similarly, the bait sequence sgA with no homology to the genomes of humans, chickens, pigs, prokaryotic DNA sequences, or viruses was cloned into PX459 to yield PX459-sgA-gRNA (36). To construct the donor plasmid pGEM-sgA-LoxP-PacI-GFP-PacI-LoxP-SfiI-VP2-SfiI-sgA containing green fluorescent protein (GFP) and IBDV (G2d) VP2 expression cassettes, sgA-LoxP-PacI-LoxP-SfiI-spacer-SfiI-sgA was synthesized by a commercial gene-synthesis service (Cosmogenetech, Seoul, Republic of Korea) and cloned into a pGEM-T-easy vector to generate pGEM-sgA-LoxP-PacI-LoxP-SfiI-spacer-SfiI-sgA. The GFP expression cassette from pEF-GFP (Addgene, Watertown, MA, USA) was cloned into pGEM-sgA-LoxP-PacI-LoxP-SfiI-spacer-SfiI-sgA via the PacI site to generate pGEM-sgA-LoxP-GFP-LoxP-SfiI-spacer-SfiI-sgA. The IBDV (G2d) VP2 expression cassette was then cloned into pGEM-sgA-LoxP-GFP-LoxP-SfiI-spacer-SfiI-sgA via SfiI sites to generate the final donor plasmid pGEM-sgA-LoxP-PacI-GFP-PacI-LoxP-SfiI-VP2-SfiI-sgA. Detailed methods for construction of Cas9/gRNA-expression plasmids and donor plasmids were previously described (34). The primer sequences used are listed in Supplementary Table 1.

### 2.3 Generation of recombinant rHVT-VP2-HA

**Figure 1:**
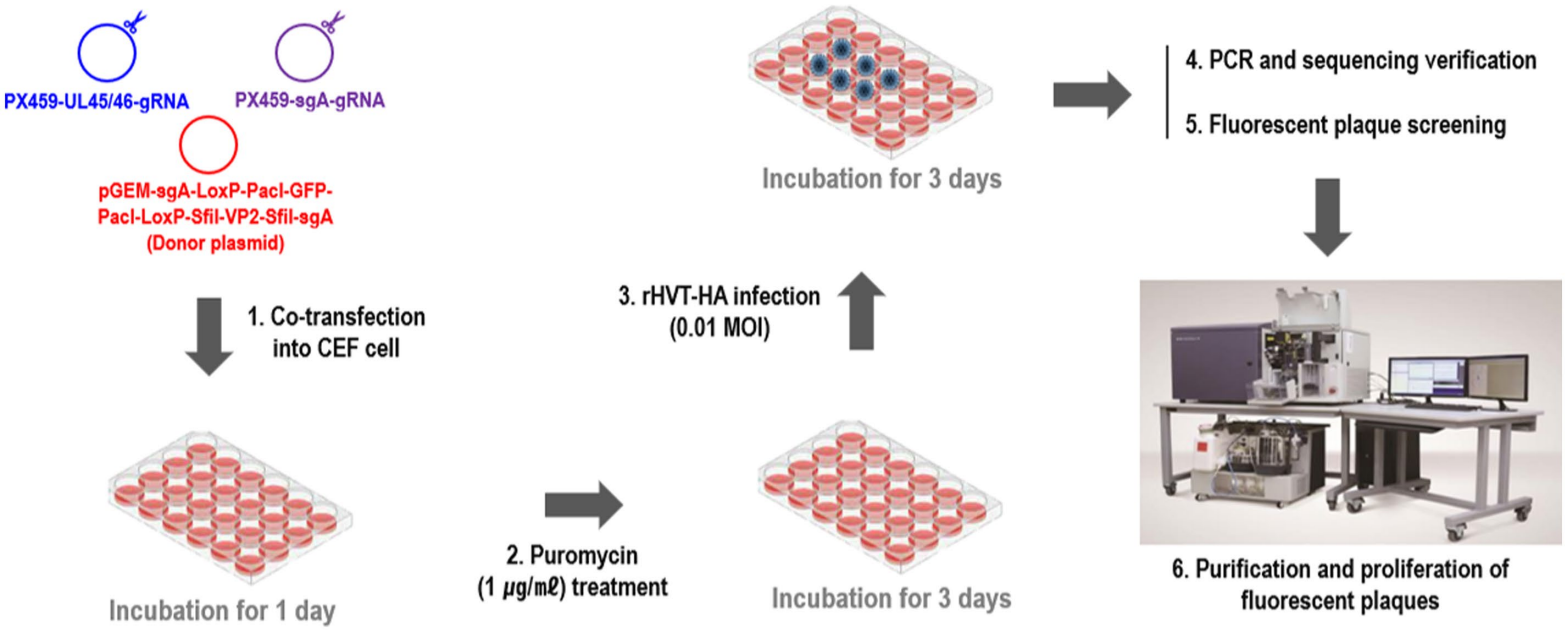
Diagram of the protocol used to generate the recombinant virus rHVT-VP2-HA.

To construct the recombinant virus rHVT-VP2-HA, NHEJ-CRISPR/Cas9 gene-editing technology was used (Supplementary Figure 1). Primary CEFs were seeded into 24-well plates 2 days before transfection at a density of 4 × 10^5^ cells/well. Then, 0.25 μg of PX459-UL45/46-gRNA, 0.25 μg of PX459-sgA-gRNA, and 0.5 μg of donor plasmid pGEM-sgA-LoxP-PacI-GFP-PacI-LoxP-SfiI-VP2-SfiI-sgA were transfected into CEFs using Lipofectamine 3000® (Invitrogen, Carlsbad, CA, USA) according to the manufacturer’s protocol. Twenty-four hours after transfection, cells were treated with puromycin for 3 days and then infected with rHVT-HA at a multiplicity of infection (MOI) of 0.01 plaque-forming units (pfu)/cell. Detailed methods for the generation of rHVT-HA that expressing H9N2/Y280-HA was previously described (35). Three days after infection, half of the cells were used for PCR identification to determine if gene editing occurred, and the other half were re-seeded in new CEFs for fluorescent plaque screening and purification (Figure 1). Fluorescence-activated cell sorters (FACS) method was used for plaque purification. rHVT-VP2-Y280 was harvested and sorted into a 96-well plate pre-seeded with CEFs using the BD FACS Aria III cell sorter (Becton, Dickinson and company, Franklin Lakes, NJ) with Cy3 and FITC excitation. RFP and GFP-positive rHVT-VP2-HA infected cells were subsequently purified using three rounds of purification, and rHVT-VP2-HA purity was checked by PCR in every round of purification. Primer sequences are detailed in Supplementary Table 1.

### 2.4 Stability

The recombinant virus rHVT-VP2-HA was grown sequentially in CEFs for 15 passages, and the presence of inserted gene (GFP-VP2) was examined by PCR using a DNA sample extracted from every fifth passage.

### 2.5 Animal experiment 1

#### 2.5.1 Chickens and vaccination programs

To test the protective efficacy of the recombinant virus rHVT-VP2-HA against IBDV (G2d), high-health status 46 one-day-old SPF chickens were distributed into five groups [A–C, positive control (PC), and negative control (NC)], and chickens were kept in temperature-controlled isolators and provided *ad libitum* access to feed and water. Group A (*n* = 10) and group B (*n* = 10) were vaccinated with rHVT-VP2-HA and commercial HVT-IBD vector vaccine (Vaxxitek), respectively, via the subcutaneous route under the skin of the neck with 6,000 PFU in a 200 μL volume. Group C (*n* = 10) was vaccinated with commercial live attenuated IBD vaccine (IBD BLEN) via the intraocular route with 2 doses. The PC (*n* = 10) and NC (*n* = 6) groups were immunized with PBS in the same way. At before challenge, three chickens from groups A, B, C, and PC were euthanized and examined for gross lesions in bursa. At 3 weeks post-vaccination (WPV), groups A, B, and C and the PC group were challenged intraocularly with 200 μL of 10^7^ EID_50_ of IBDV (G2d). At 7 days post-infection (DPI), all survived chickens were humanely euthanized by CO_2_ asphyxiation, necropsied, and examined for the presence of gross lesions. Furthermore, bursae were collected for the virus detection. Vaccine protection indices (PI) were calculated using the following formula: [PI = 100% – (Bursal atrophy rate of the vaccinated group)/(Bursal atrophy rate of the sham group)].

All experimental and animal management procedures were undertaken to ensure animal health and well-being throughout the study. Additionally, the study was approved by and in accordance with the requirements of the Animal Care and Ethics Committee of Jeonbuk National University. The animal facility at Jeonbuk National University is fully accredited by the National Association of Laboratory Animal Care (approval number: NON2023-007).

#### 2.5.2 Serology

Blood samples were collected from each group at 1, 2, and 3 WPV. The samples were placed at 37°C for 1 h, followed by centrifugation at 3,000 rpm for 15 min to separate the sera. IBD-antibody titers were measured using a commercial VDPro® IBDV AB ELISA kit (Median Diagnostics). Based on the manufacturer’s instructions, serum samples with the ELISA antibody titer ≥750 were considered positive.

#### 2.5.3 Clinical signs, mortality, and postmortem lesions

The birds were checked daily for mortality and clinical signs within a week after challenge. Dead birds were dissected and examined for gross lesions. At 7 DPI, all surviving birds were subjected to autopsy, and the gross lesions were examined.

#### 2.5.4 Bursa to body weight ratio (b/b ratio), bursal body weight index (BBIX), spleen to body weight ratio (s/b ratio) and histopathology

The bursa weight, spleen weight, and body weight were recorded for each bird. The bursa to body weight ratio (b/B ratio) was calculated using the following formula: [b/B ratio = (bursa weight)/(body weight) × 1,000]. The bursal-body weight index (BBIX) was calculated as follows: [BBIX = (b/B ratio in the infected group)/(b/B ratio in the control group)]. A BBIX less than 0.7 was considered atrophy. The spleen to body weight ratio (s/B ratio) was calculated as follows: [s/B ratio = (spleen weight)/(body weight) × 1,000].

#### 2.5.5 Reverse transcription PCR (RT-PCR)

Viral RNA was extracted from the clarified bursal samples using the 5X MagMAX™ – Pathogen RNA/DNA kit (Thermo Fisher Scientific, Vilnius, Lithuania) with the KingFisher Duo Prime Purification system (Thermo Fisher Scientific, Waltham, MA, USA) following the manufacturer’s protocol. Viral cDNA was generated from RNA samples using GoScript™ reverse transcriptase (Promega, Madison, WI USA) with random primers (9-mers; TaKaRa Bio. Inc., Otsu, Shiga, Japan). In the reverse transcription (RT) reaction, 8 μL of extracted RNA and 2 μL of dimethyl sulfoxide (Tedia, Ohio, USA) were heated at 100°C for 5 min and then placed in an ice bath for 5 min. Then, the following materials were added to this reaction mixture: 8 μL of GoScript™ 5X RT reaction buffer (Promega, Madison, WI, USA), 10 μL of 2.0 mM dNTP (SolGent, Daejeon, Korea), 4 μL of MgCl_2_ (Promega, Charbonnie‘re, France), 1 μL of 20 units Recombinant RNasin® Ribonuclease Inhibitor (Promega, Madison, WI, USA), 1 μL of GoScript™ reverse transcriptase, 1 μL of 50 pmol random primer, and 4 μL of diethylpyrocarbonate-treated water (DEPC water; Biosesang, Seoul, Korea); a final volume of 39 μL was obtained. The RT reaction mixture was incubated in this sequence: 25°C for 5 min, 42°C for 60 min, and 70°C for 15 min to inactivate the enzyme (41). RT-PCR was performed using the forward primer IBDV-F1 (5’-GCCCAGAGTCTACACCAT-3’) and the reverse primer IBDV-R1 (5’-CCCGGATTATGTCTTTGA-3’), which amplified a 743 bp fragment covering the hypervariable region (HVR) of the VP2 gene (42).

### 2.6 Animal experiment 2

#### 2.6.1 Chickens and vaccination programs

To test the protective efficacy of the recombinant virus rHVT-VP2-HA against H9N2/Y280, high-health status 44 one-day-old SPF chickens were divided into six groups [A–D, positive control (PC), and negative control (NC)], and chickens were kept in temperature-controlled isolators and provided *ad libitum* access to feed and water. Group A (*n* = 10) and group B (*n* = 8) were immunized with a 200-μl volume of 6,000 PFU of rHVT-VP2-HA and rHVT-HA, respectively, via the subcutaneous route under the skin of the neck. Group C (*n* = 8) was vaccinated with 0.1% formalin inactivated H9N2/Y280 via the intramuscular route with 10^8.5^ EID_50_/0.1 mL. Group D (*n* = 8) was vaccinated with commercial inactivated H9N2/Y439 vaccine (based on A/chicken/Korea/01310/2001 strain) via the intramuscular route with 1 dose. The PC (*n* = 8) and NC (*n* = 2) groups were immunized via the subcutaneous route under the skin of the neck with 200 μL of PBS. At 21 DPV, groups A, B, C, and D and the PC group were challenged intranasally with 400 μL volume of 10^7.3^ EID_50_ of H9N2/Y280. Oropharyngeal (OP) and cloacal (CL) swabs were collected at 3 and 5 DPI to assess virus shedding. At the end of experiments, all survived chickens were humanely euthanized by CO_2_ asphyxiation, and tissue samples [lung, tracheal, cecal tonsil (CT), brain, and bursa] were collected at 5 DPI for viral detection. All experimental and animal management procedures were undertaken to ensure animal health and well-being throughout the study. Additionally, the study was approved by and in accordance with the requirements of the Animal Care and Ethics Committee of Jeonbuk National University. The animal facility at Jeonbuk National University is fully accredited by the National Association of Laboratory Animal Care (approval number: NON2023-007).

#### 2.6.2 Serology

Blood samples were collected from each group at 1, 2, and 3 WPV. The samples were placed at 37°C for 1 h, followed by centrifugation at 3,000 rpm for 15 min to separate the sera. Serum antibody titers against HA were quantified using the haemagglutinin inhibition (HI) assay according to standard protocol (43). Briefly, two-fold dilutions of chicken serum samples were tested in duplicate in 96-well V-bottomed plates, followed by adding 4 hemagglutination units (HAU) of antigens (H9N2/Y280 and H9N2/Y439) that genetically and antigenically surrogate for the used vaccine strains and diluting in PBS. After the plates were incubated at room temperature for 0.5 h, 0.5% chicken red blood cells were added to the virus/serum mixture and incubated at room temperature for another 30 min. The HI antibody titer was determined as the reciprocal of the highest dilution that completely prevented red blood cells from agglutination.

#### 2.6.3 Assessment of virus shedding

OP and CL swabs were suspended in 1 mL of PBS containing 1% antibiotic-antimycotic solution (Gibco, New York, USA) and kept at −70°C until use. RNA was extracted using the QIAamp viral RNA isolation kit (Qiagen, Valencia, CA, USA) according to the manufacturer’s instructions. The viral titer of each sample was determined using commercial qRT-PCR kit, VDx® AIV qRT-PCR kit (Median Diagnostics, Seoul, Republic of Korea) that targeting AIV-M gene. To convert the Ct values to EID_50_ equivalents/0.1 mL, quantitative viral standards (ranging from 10^7.0^ to 10^0.0^ EID_50_/0.1 mL) of the virus were prepared. Viral RNA was extracted and quantified by qRT-PCR. The resulting standard curve demonstrated a strong correlation (*r*^2^ > 0.99) and was used to convert Ct values to EID_50_ equivalents/0.1 mL. The detection limit was 10^1.0^ EID_50_ /0.1 mL, with a Ct value of 37.

#### 2.6.4 Virus replication in tissues

The tissue samples (lung, tracheal, CT, brain, and bursa) were homogenized in 10% (w/v) PBS (pH 7.4; supplemented with 100× antibiotic-antimycotic solution). The homogenates were centrifuged at 3,000 × *g* for 10 min at 4°C, and the supernatant was then conserved in aliquots at −70°C for qRT-PCR.

### 2.7 Statistical analysis

All statistical analysis was performed using SPSS version 21.0 (SPSS Inc., Chicago, IL, USA). The number of chickens was estimated by a power analysis comparing the size of the difference in the variable interest between vaccinated and control groups based on either previous or pilot experiments. By using 80% power, *p* = 0.05, and a two-tailed t test, minimum numbers of chickens were chosen and used in this study. Statistical differences within experiments were analyzed and compared by Student’s *t*-test, one-way analysis of variance (ANOVA) or Kruskal–Wallis all pair-wise comparison test. Group means were compared with Fisher least significant difference (LSD) test. The data were analyzed by Differences were considered statistically significant at ∗*p* < 0.05, ∗∗*p* < 0.01, and ∗∗∗*p* < 0.001.

## 3 Results

### 3.1 Generation of recombinant virus rHVT-VP2-HA

**Figure 2:**
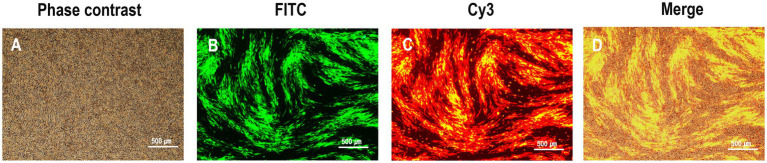
Observation of plaques expressing both green and red fluorescence during rHVT-VP2-HA screening. rHVT-VP2-HA-infected CEFs under phase contrast **(A)**, and FITC excitation **(B)**, and Cy3 excitation **(C)**, and merge of both **(D)**.

To generate the recombinant virus rHVT-VP2-HA, CEFs were co-transfected with PX459-UL45/46-gRNA, PX459-sgA-gRNA, and donor plasmid pGEM-sgA-LoxP-PacI-GFP-PacI-LoxP-SfiI-VP2-SfiI-sgA. At 24-h post-transfection, the cells were treated with puromycin for 3 days and then infected with rHVT-HA at an MOI of 0.01. At 3 DPI, fluorescent plaques expressing GFP and RFP appeared, indicating that the donor sequence GFP-VP2 had been successfully inserted into the rHVT-HA genome (Figure 2). Four pairs of primers were used for PCR identification. The primers UL45-F/VP2-R and G2d-3F/UL45/46-R with target bands of 2,633 bp and 548 bp, respectively, were used to identify viruses inserted in the forward orientation (Supplementary Figure 1). The primers UL45-F/G2d-3F and VP2-R/UL45/46-R with target bands of 589 bp and 2,592 bp, respectively, were used to identify viruses inserted in the reverse orientation (Supplementary Figure 2). The electrophoresis results were as expected (Supplementary Figure 3). The sequencing results were also consistent with the expected results (Supplementary Figures 4–7). In summary, GFP-VP2 was inserted into the rHVT-HA genome in both forward and reverse orientations.

### 3.2 Purification of recombinant virus rHVT-VP2-HA

**Figure 3:**
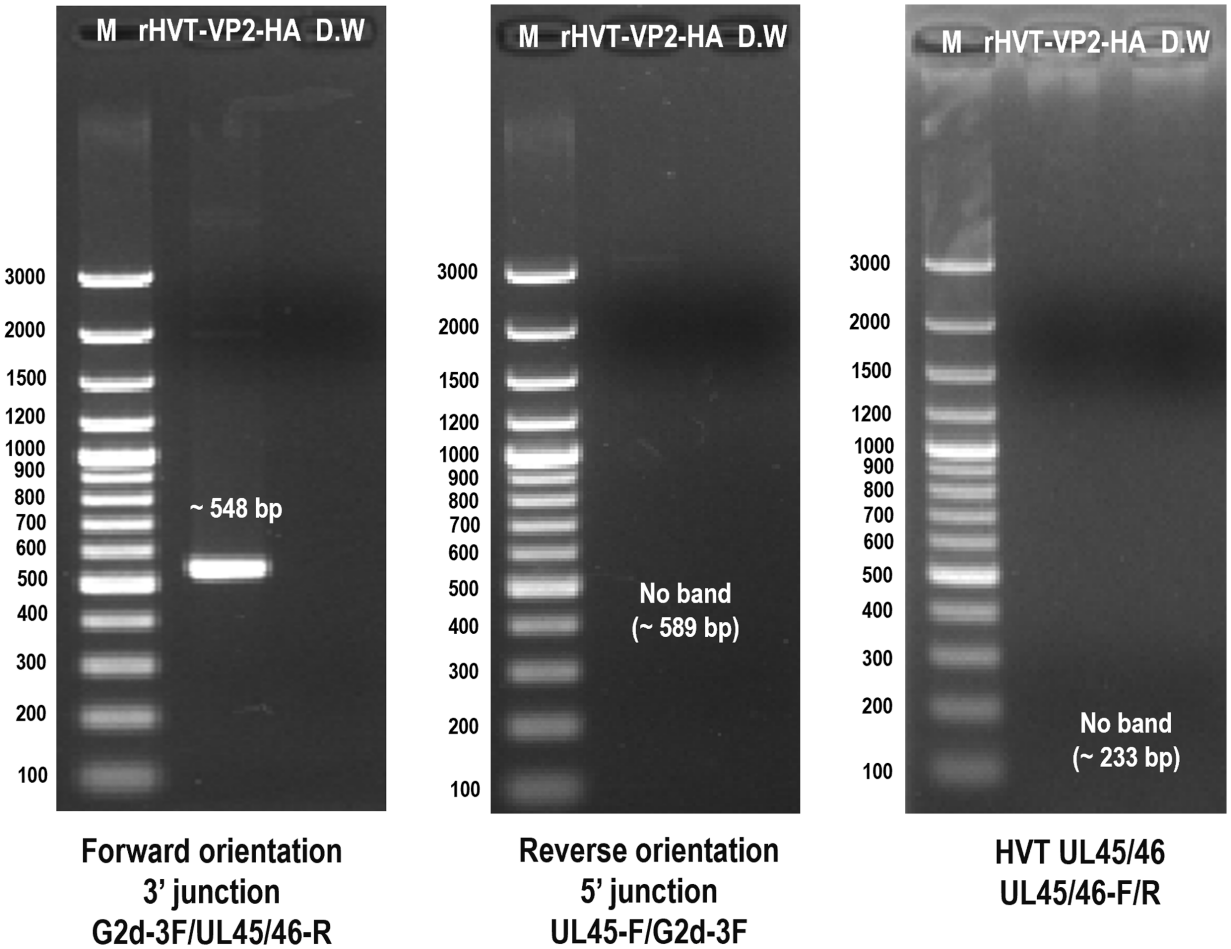
Identification of purified recombinant virus rHVT-VP2-HA by PCR. The primers G2d-3F/UL45/46-R were used to identify the virus inserted in the forward orientation. The primers UL45-F/G2d-3F were used to identify the virus inserted in the reverse orientation. The primers UL45/46-F/UL45/46-R were used to detect the presence of HVT.

After three rounds of purification, the recombinant virus rHVT-VP2-HA was identified by PCR. The primers G2d-3F/UL45/46-R were used to identify the virus inserted in the forward orientation. The target band was 548 bp. The primers UL45-F/G2d-3F were used to identify the virus inserted in the reverse orientation. The target band was 589 bp. The primers UL45/46-F/UL45/46-R were used to detect the presence of HVT or not. The target band was 233 bp bigger amplicon (~5 kb) for inserted expression cassette was not amplified by primer set (UL45/46-F/UL45/46-R), it may relate to cycle conditions that may not be sufficient for amplification of bigger amplicon. The results showed that GFP-VP2 was inserted in the forward orientation in the finally purified recombinant virus (Figure 3).

### 3.3 Stability

The genetic stability of the GFP-VP2 and RFP-HA genes were measured by passing rHVT-VP2-HA sequentially in CEFs for 15 passages. After every five passages, the viral DNA was extracted and analyzed by PCR. The PCR results showed that the target band was amplified from the 5th, 10th, and 15th passages of the rHVT-VP2-HA DNA samples. These results indicated the stable integration of the GFP-VP2 and RFP-HA genes in the HVT genome even after 15 passages.

### 3.4 Experiment 1

#### 3.4.1 Immune response

**Figure 4:**
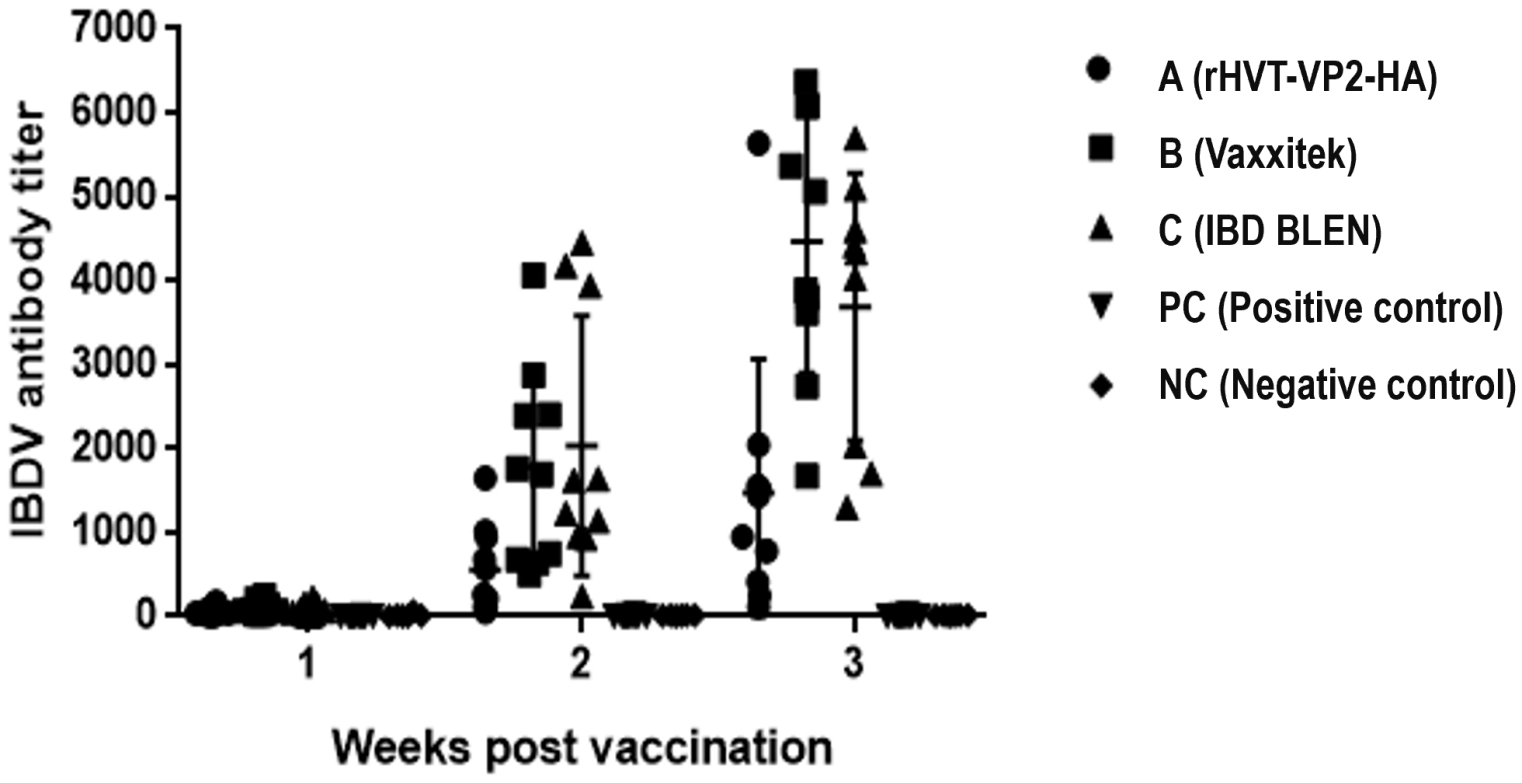
Detection of IBDV antibody titers of immunized chickens by ELISA. Sera were collected weekly and detected using a commercial VDPro® IBDV AB ELISA kit.

To assess the immunogenicity of rHVT-VP2-HA in chickens, sera were collected weekly and checked by ELISA after vaccination. According to the manufacturer’s instructions, immune status was considered positive if the ELISA titer was above 750. At 2 WPV, the seropositivity rates of groups A (rHVT-VP2-HA), B (Vaxxitek), and C (IBD BLEN) were 30, 60, and 90%, respectively, with antibody titers of 559 ± 518, 1775 ± 1,174, and 2033 ± 1,543, respectively. At 3 WPV, the positive rates of groups A, B, and C were 70, 100, and 90%, respectively, and the mean antibody titers were 1,470 ± 1,595, 4,467 ± 1,572, and 3,393 ± 1773, respectively. The average antibody level induced by rHVT-VP2-HA was higher than 750. These results suggest that although it induced lower levels of antibodies than the commercial vaccines Vaxxitek and IBD BLEN, rHVT-VP2-HA can induce a high level of humoral immunity (Figure 4).

#### 3.4.2 Clinical signs, mortality, pathology, and virus detection

No clinical signs or mortality were recorded in all groups during the 7 days observation period after challenge. All chickens were necropsied at 7 DPI. The NC group did not show any gross lesions. In the PC group, one of the eight chickens showed hemorrhage of the thigh muscle. The main gross lesion detected in the chickens was atrophy of the bursa (Table 1). In the PC group and group C (IBD BLEN), all birds showed bursal atrophy. In group B (Vaxxitek), 29% (2/7) of chickens showed bursal atrophy. However, none of the chickens in group A (rHVT-VP2-HA) showed bursal atrophy. The results for the b/B ratio and BBIX were also consistent with those for bursal lesions. The b/B ratios of the NC group and group A were 4.34 ± 0.53 and 4.85 ± 1.04, respectively, with no statistically significant difference. However, the b/B ratios of the PC group and group C were statistically significantly lower at 1.69 ± 0.38 and 1.25 ± 0.34, respectively (*p* < 0.05). The b/B ratio of group B (3.93 ± 1.34) was also lower than that of the NC group (4.34 ± 0.53). Group A had the highest BBIX of 1.12 ± 0.24. The PC group and group C had the lowest BBIX values of 0.39 ± 0.09 and 0.29 ± 0.08, respectively, and that of group B was in between (0.91 ± 0.31). Regarding the s/B ratio, there was no significant difference between the NC group and group A, but the values in the PC group, group B, and group C were significantly increased (*p* < 0.05), indicating that the spleens of chickens in these three groups had a certain degree of swelling.

**Table 1:** Protective efficacy against IBDV (G2d) challenge.

Group	Before challenge (3 WPV)		7 DPI							
	b/B ratio	BBIX^a^	Mortality	Gross lesions^1^	Bursa lesions^2^	b/B ratio^3^	BBIX^4^ (atrophy rate)	s/B ratio^5^	PCR (Bursa)	PI^6^
A (rHVT-VP2-HA)	5.04 ± 0.79^a^	0.93 ± 0.15	0/7 (0%)	0/7 (0%)	0/7 (0%)	4.85 ± 1.04^a^	1.12 ± 0.24 (0/7, 0%)	2.04 ± 0.36^a^	0/7 (0%)	100%
B (Vaxxitek)	5.05 ± 0.69^a^	0.93 ± 0.13	0/7 (0%)	0/7 (0%)	2/7 (29%)	3.93 ± 1.34^b^	0.91 ± 0.31 (2/7, 29%)	2.34 ± 0.41^b^	2/7 (29%)	71%
C (IBD BLEN)	1.34 ± 0.13^b^	0.25 ± 0.13	0/7 (0%)	0/7 (0%)	7/7 (100%)	1.25 ± 0.34^c^	0.29 ± 0.08 (7/7, 100%)	2.28 ± 0.68^b^	2/7 (29%)	0%
PC (Positive control)	5.44 ± 1.26	–	0/7 (0%)	1/7 (14%)	7/7 (100%)	1.69 ± 0.38^c^	0.39 ± 0.09 (7/7, 100%)	2.70 ± 0.35^c^	7/7 (100%)	–
PC (Negative control)	–	–	0/6 (0%)	0/6 (0%)	0/6 (0%)	4.34 ± 0.53^a^	(0/7, 0%)	1.85 ± 0.27^a^	0/6 (0%)	–

1 Gross lesions: Leg muscle hemorrhage, kidney swelling and hemorrhage, proventricular hemorrhage.

2 Bursal lesions: Bursal hemorrhage, inflammation, atrophy.

3 b/B ratio = Bursa weight/Body weight × 1,000.

4 BBIX = (b/B ratio in the infected group)/(b/B ratio in the control group). A BBIX value less than 0.7 was considered to indicate atrophy.

5 s/B ratio = Spleen weight/Body weight × 1000.

6 PI: protection index = 100% – Bursal atrophy rate of the vaccinated group/Bursal atrophy rate of the sham group.

^abc^Means *p* < 0.05.

**Figure 5:**
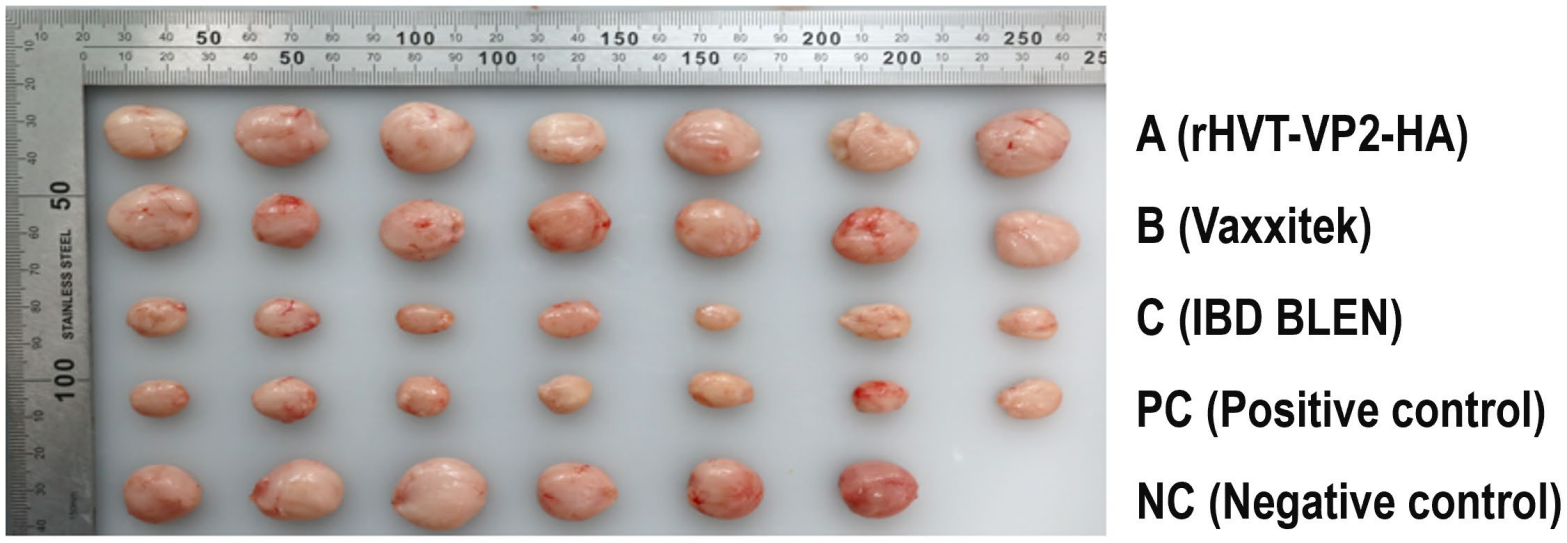
Size and morphology of the bursa of Fabricius recovered from the chickens at 7 DPI with the IBDV (G2d) strain. Chickens in groups A, B, and C were inoculated subcutaneously with rHVT-VP2-HA, Vaxxitek, and IBD BLEN, respectively, at doses of 6,000 PFU. Chickens in the PC and NC groups were inoculated subcutaneously with 0.2 ml of PBS as controls.

Since the main damage caused by IBDV (G2d) in chickens is bursal atrophy, we calculated the PI based on the atrophy rate of the bursa. The PIs of group A, group B, and group C were 100, 71 and 0%, respectively. In addition, the bursae were also tested for IBDV to determine whether IBDV was present and to confirm whether the virus was the strain used for the challenge or the vaccine. The results showed that no IBDV was detected in group A, and only the challenge strain IBDV (G2d) was detected in groups B and C and the PC group, with detection rates of 29% (2/7), 29% (2/7), and 100% (7/7), respectively. These results indicated that rHVT-VP2-HA could provide 100% protection against IBDV (G2d) infection, while the commercial vaccine Vaxxitek only provided partial protection (Figure 5).

### 3.5 Animal experiment 2

#### 3.5.1 Humoral immune response

To assess the immunogenicity of rHVT-VP2-HA in chickens, sera were collected weekly and checked by HI after vaccination. At 2 WPV, the seropositivity rates of group A (rHVT-VP2-HA), group B (rHVT-HA), group C (Inactivated H9N2/Y280), and group D (Inactivated H9N2/Y439) were 60, 100, 100, and 100%, respectively, with antibody titers of 2.20 ± 2.30, 4.63 ± 1.06, 6.75 ± 1.04, and 3.88 ± 0.64, respectively. At 3 WPV, the positive rates of groups A, B, C, and D were all 100%, with mean antibody titers of 7.10 ± 1.52, 6.88 ± 0.99, 10.00 ± 0.76, and 6.25 ± 0.89, respectively. These results suggest that rHVT-VP2-HA can induce a high level of humoral immunity.

#### 3.5.2 Protective efficacy of rHVT-VP2-HA in chickens

No clinical signs or mortality were recorded in all groups during the 5 days observation period after challenge. All chickens were necropsied at 5 DPI (Table 2). Gross lesions were the most severe in the PC group. Chickens in the PC group showed hemorrhagic tracheas (4/8), hemorrhagic thymuses (3/8), swollen kidneys (4/8), and hemorrhagic bursae (1/8). Gross lesions in group D (Inactivated H9N2/Y439) were next. Chickens in group D showed hemorrhagic tracheas (4/8), hemorrhagic thymuses (3/8), and swollen kidneys (3/8). Chickens in group A (rHVT-VP2-HA), group B (rHVT-HA), group C (Inactivated H9N2/Y280), and the NC group did not show any gross lesions. At 3 DPI, the virus shedding rate was the highest in the PC group at 100% (8/8) and 75% (6/8) in OP and CL samples, respectively. In group D, the virus shedding rates were 100% (8/8) and 25% (2/8) in OP and CL samples, respectively. Viruses were not detected in OP and CL samples from groups A, B, and C.

**Table 2:** Protective efficacy against H9N2/Y280 challenge.

Group	Serology at 3 WPV (log_2_ HI titer)^a^	Virus shedding (log_10_EID_50_/0.1 mL)^b^				Virus replication in tissues (log_10_EID_50_/0.1 mL)^c^					
		3 DPI		5 DPI		5 DPI					PI^d^
		OP	CL	OP	CL	Trachea	Lung	Brain	Bursa	Cecal Tonsil	
A (rHVT-VP2-HA)	7.1 (10/10)	0/10 (0)	0/10 (0)	0/10 (0)	0/10 (0)	0/10 (0)	1/10 (0.1)	0/10 (0)	0/10 (0)	0/10 (0)	100%
B (rHVT-HA)	6.9 (8/8)	0/8 (0)	0/8 (0)	0/8 (0)	0/8 (0)	0/8 (0)	0/8 (0)	0/8 (0)	0/8 (0)	0/8 (0)	100%
C (Inactivated H9N2/Y280)	10.0 (8/8)	0/8 (0)	0/8 (0)	0/8 (0)	0/8 (0)	2/8 (0.5)	0/8 (0)	0/8 (0)	0/8 (0)	0/8 (0)	100%
D (Inactivated H9N2/Y439)	6.3 (8/8)	8/8 (3.9)	2/8 (0.6)	8/8 (2.3)	2/8 (0.4)	7/8 (2.6)	4/8 (1.2)	3/8 (0.6)	3/8 (0.9)	2/8 (0.3)	50%
PC (Positive control)	0 (0/8)	8/8 (4.2)	6/8 (1.1)	8/8 (3.2)	6/8 (1.8)	8/8 (4.2)	6/8 (1.5)	5/8 (1.1)	4/8 (1.0)	4/8 (1.1)	–
NC (Negative control)	0 (0/2)	0/2 (0)	0/2 (0)	0/2 (0)	0/2 (0)	0/2 (0)	0/2 (0)	0/2 (0)	0/2 (0)	0/2 (0)	–

a No. serology positive/total survived in group (mean HI titer).

b No. virus positive/total in group.

c Tissue samples were taken at 5 dpi; virus positive/total in group (virus titers of the pooled samples).

d Inhibition of virus recovery rate against the number of positive detections in the cecal tonsils = 100% – positive detection rate of the vaccinated group/positive detection rate of the sham group.

At 5 DPI, the virus shedding rate in the PC group was still high at 100% (8/8) and 75% (6/8) in OP and CL samples, respectively. In group D, the virus shedding rates in OP and CL samples were 100% (8/8) and 25% (2/8), respectively. However, in OP and CL samples from group A, B, and C, no viruses were detected. To evaluate virus replication in tissues, tracheal, lung, brain, bursal and CT tissues were collected at 5 DPI. For the PC group, the virus detection rates in tracheal, lung, brain, bursal and CT tissues were 100% (8/8), 75% (6/8), 62.5% (5/8), 50% (4/8), and 50% (4/8), respectively, and those in group D were 87.5% (7/8), 50% (4/8), 37.5% (3/8), 37.5% (3/8), and 25% (2/8), respectively. However, the virus detection rates in groups A, B, and C were very low. In group A, the virus was only detected in lung tissues, and the detection rate was only 10% (1/10). In group B, the virus was not detected in tissue samples. In group C, the virus was only detected in tracheal tissues with a detection rate of 25% (2/8). The virus was not detected in all OP and CL samples and tissue samples in the NC group.

According to the virus detection rate in CT tissues, the calculated PIs of groups A, B, C, and D were 100, 100, 100, and 50%, respectively. Overall, the above results show that rHVT-VP2-HA, rHVT-HA and inactivated H9N2/Y280 can provide 100% protection against H9N2/Y280; however, inactivated H9N2/Y439 could only provide partial protection.

## 4 Discussion

Vaccination is widely used to control infections that severely impact the economics of the poultry industry. Vaccines will play a bigger role in health management programs for the protection of poultry flocks in the entire industry as there is a greater emphasis on avoiding the use of antibiotics in poultry production systems. Since chickens need to receive many different vaccines to prevent many poultry diseases, it is essential to develop multivalent vaccinations that can simultaneously provide protection against multiple avian diseases. HVT is the most successful recombinant viral vector ever developed. It is widely used commercially to deliver various protective antigens for the prevention and control of poultry diseases such as NDV, IBDV, and AIV (44).

However, when different HVT-vectored vaccines are used in combination, there will be interference between the vaccines; thus, the expected immune effect cannot be achieved (45, 46). Therefore, the combined use of different HVT-vectored vaccines is limited.

Recently, some scholars have successfully integrated multiple foreign genes into the HVT genome using CRISPR/Cas9 gene editing technology, which prompted us to use the same strategy to insert protective antigens of new viruses to develop multivalent recombinant HVT-vectored vaccines (47, 48). In previous studies, we have constructed two single-insertion recombinant viruses: rHVT-VP2 and rHVT-HA. The VP2 gene was from IBDV (G2d), and the HA gene was from H9N2/Y280 (34, 35). Their vaccine efficacy has been evaluated separately. The results showed that rHVT-VP2 and rHVT-HA can produce complete protection against the new variant IBDV (G2d) and the newly identified H9N2/Y280, respectively, and both of them have potential as vaccine candidates. Multivalent vaccines are an inevitable trend in vaccine development. A multivalent vaccine not only reduces the number of injections and simplifies the immunization procedure but is also convenient for poultry farmers. The purpose of this study was to simultaneously insert the VP2 gene of IBDV (G2d) and the HA gene of H9N2/Y280 into the HVT genome to construct a bivalent vaccine with HVT as the vector and to test whether the efficacy of this bivalent vaccine is equivalent to that of the two monovalent vaccines.

The constant emergence of new variants of infectious poultry viruses requires the poultry industry to be able to rapidly develop corresponding vaccines. The main methods for constructing HVT-vectored recombinant vaccines include traditional homologous recombination, the bacterial artificial chromosome (BAC) method, and the overlapping cosmid system, but these methods are time consuming and labor intensive and cannot meet the needs of modern vaccine development (23). CRISPR/Cas9 is a third-generation gene editing technology that is simple, fast, and efficient. The CRISPR/Cas9 system only needs sgRNA and a donor plasmid carrying the target gene and can quickly and accurately cut specific sites to achieve efficient knock-in of the target gene (36, 49). CRISPR/Cas9 system has been widely applied in vaccine development for its high efficiency, specificity, flexibility, simplicity, and low cost compared to the other methods, which has demonstrated an efficient strategy for future development of genetically engineered vaccines (50).

Therefore, it is an important goal to establish a rapid development platform for HVT-vectored vaccines based on CRISPR/Cas9 to face the threat of any novel poultry variant viruses.

Regarding the screening of recombinant viruses, GFP and RFP are good selection markers. Plaques that can emit both green and red fluorescence were our target for recombinant virus rHVT-VP2-HA. Through this feature, the recombinant virus was quickly identified. After three rounds of plaque purification, purified viruses were obtained. Moreover, we also tried to use the FACS method to purify the recombinant virus. Through FACS, we obtained many single cells that could simultaneously emit green and red fluorescence and expanded the culture of these single cells to quickly obtain the purified target virus. These results indicate that FACS is an effective method to purify the recombinant virus rHVT-VP2-HA. In addition, GFP and RFP are only marker genes for screening recombinant viruses (34, 35, 47). LoxP and LoxN sites are designated at both ends of GFP and RFP, respectively, and these marker genes can be easily removed by Cre recombinase.

In animal experiment 1, we verified the protective efficacy of the double-insertion recombinant virus rHVT-VP2-HA against IBDV (G2d). Our research results showed that rHVT-VP2-HA could stimulate the body to produce specific antibodies against VP2 and could provide 100% protection to chickens after IBDV (G2d) challenge at 3 WPV. The bursae of chickens in the rHVT-VP2-HA group were completely normal, while all the chickens in the PC group showed atrophy with splenomegaly. However, the Vaxxitek vaccine control group showed partial atrophy with splenomegaly, demonstrating that this vaccine did not provide 100% protection to chickens. It may relate to antigenic differences of inserted VP2 in HVT-vectored vaccine. Inserted VP2 from the Vaxxitek is cvIBDV (VP2) and rHVT-VP2-HA is IBDV (G2d), respectively. In previous study, obvious antigenic mismatch between newly emerging IBDV (G2d) and commercial vaccine strain were existed, and commercial vaccine can provide partial protection against IBDV (G2d) in chickens. These results suggested that development of antigen-matched vaccine is important for the control of IBDV (G2d) (51). In addition, compared with our previous experimental results, the efficacy of the bivalent vaccine rHVT-VP2-HA against IBDV (G2d) was found to be equivalent to that of the monovalent vaccine rHVT-VP2 (34). In animal experiment 2, we verified the protective efficacy of the double-insertion recombinant virus rHVT-VP2-HA against H9N2/Y280. Our results showed that 3 weeks after vaccination, rHVT-VP2-HA could stimulate the body to produce specific antibodies against HA; thus, rHVT-VP2-HA provided 100% protection to chickens after H9N2 Y280 challenge. At 3 DPI and 5 DPI, H9N2/Y280 virus was completely undetectable in the OP and CL samples from chickens in the rHVT-VP2-HA group. No virus was detected in the tracheas, brains, bursae, or CTs, and only one of the lung samples (1/10) had the virus detected. The PI of the rHVT-VP2-HA group was 100%. However, virus shedding was still detectable in most OP and CL samples in the PC group, and virus replication was also detected in more than half of the tissues. The PI of the commercial inactivated H9N2/Y439 vaccine control group was only 50%. Furthermore, we found that the bivalent vaccine rHVT-VP2-HA was as effective against H9N2/Y280 as the monovalent vaccine rHVT-HA (35). Our results in animal experiment 1 and 2 suggested that multivalent rHVT-VP2-HA can provide complete protection against both of IBDV (G2d) and H9N2/Y280. Although our results shown that rHVT-VP2-HA provide complete protection, has a limitation on the short-term evaluation. According to regulatory authorities for animal vaccine production in Korea, vaccine efficacy against LPAI H9N2 was calculated on the 5 DPI in present study (52). However, H9N2/Y280 infected chickens can shed the virus over the 7 days in previous studies (17, 53). For the evaluation of protective efficacy in long-term evaluation, further studies are required. Generally, recombinant HVT vector vaccine can overcome the interference of passive immunity, such as maternal derived antibody (MDA) in commercial chickens (54, 55). However, this study was only focused on the vaccine efficacy in SPF chickens without presence of the MDA. To evaluate applicability of the rHVT-VP2-HA in the field conditions, further studies are required to evaluation of the vaccine efficacy in the commercial chickens with presence of MDA such as layers, broilers, and breeders.

Regarding the selection of insertion sites, in this study, we inserted the VP2 gene of IBDV (G2d) into the UL45/46 region and the HA gene of H9N2/Y280 into the US2 region. Many references have demonstrated that UL45/46 and US2 are good regions for inserting foreign genes. Insertion of foreign genes into these two regions does not alter the replication ability of the viruses (22, 56, 57). Studies have shown that the insertion site is an important factor that affects the expression of foreign genes. The genome of HVT is large and contains a large number of non-essential regions. To better express foreign genes and obtain vaccines with higher efficacy, our next research step is to explore and test non-essential regions. The discovery of more effective insertion sites will lay a solid foundation to construct multivalent vaccines.

## 5 Conclusion

In conclusion, the recombinant virus rHVT-VP2-HA was successfully constructed using NHEJ-CRISPR/Cas9 gene-editing technology. Vaccination of SPF chickens with rHVT-VP2-HA produced high levels of specific antibodies against IBDV (G2d) and H9N2/Y280. rHVT-VP2-HA provided 100% protection against challenges with the IBDV (G2d) and H9N2/Y280. These results demonstrate that rHVT-VP2-HA is a safe and highly efficacious vaccine for simultaneous control of IBDV (G2d) and H9N2/Y280.

## Data availability statement

The original contributions presented in the study are included in the article/Supplementary material, further inquiries can be directed to the corresponding author/s.

## Ethics statement

The animal study was approved by all experimental and animal management procedures were undertaken in accordance with the requirements of the Animal Care and Ethics Committee of Jeonbuk National University. The animal facility at Jeonbuk National University is fully accredited by the National Association of Laboratory Animal Care (approval number: NON2023-007). The study was conducted in accordance with the local legislation and institutional requirements.

## Author contributions

J-FZ: Writing – review & editing, Writing – original draft, Methodology, Data curation. KS: Writing – review & editing, Writing – original draft, Investigation, Formal analysis. S-WK: Writing – review & editing, Writing – original draft, Methodology, Data curation. J-YP: Writing – review & editing, Writing – original draft, Investigation. BW: Writing – review & editing, Writing – original draft, Validation, Supervision. H-KJ: Writing – review & editing, Writing – original draft, Supervision, Funding acquisition. MK: Writing – review & editing, Writing – original draft, Resources, Funding acquisition. S-YC: Writing – review & editing, Writing – original draft, Visualization, Supervision, Resources, Project administration, Investigation, Data curation, Conceptualization.
