# Supplementary material for: Simultaneous construction strategy using two types of fluorescent markers for HVT vector vaccine against infectious bursal disease and H9N2 avian influenza virus by NHEJ-CRISPR/Cas9
Source: Front Vet Sci. 2024 May 13;11:1385958. doi: 10.3389/fvets.2024.1385958 (PMC11135205; doi:10.3389/fvets.2024.1385958)
Supplement: Supplementary file 1 [file Data_Sheet_1.PDF]

Supplementary Material

1 Supplementary Figures

1.1 Supplementary Figure 1

PCR identification of the recombinant virus rHVT-VP2-HA with GFP-VP2 forward insertion. The primers UL45-F/VP2-R and G2d-3F/UL45/46-R were used to identify viruses inserted in the forward orientation, with target bands of 2,633 bp and 548 bp, respectively.

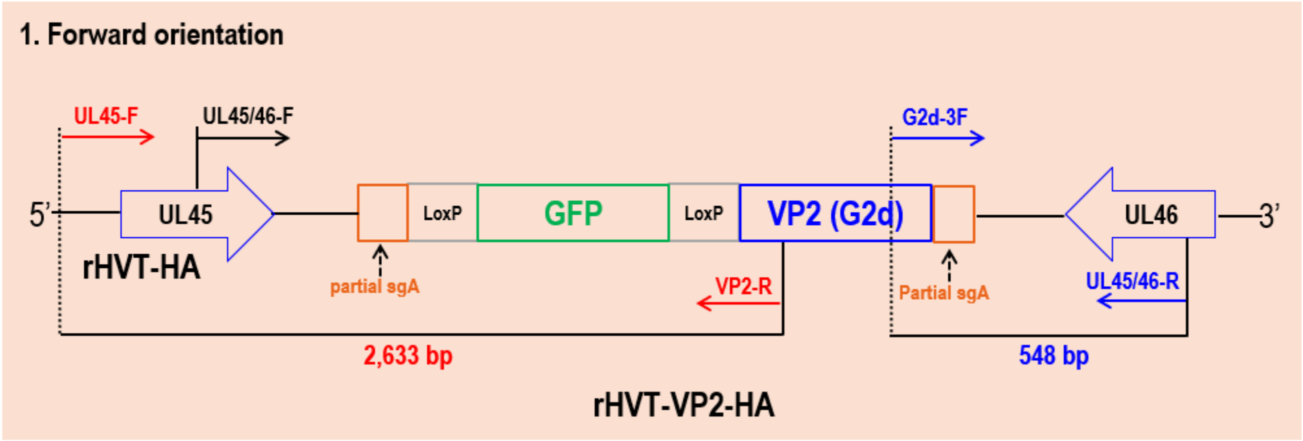

1.2 Supplementary Figure 2

PCR identification of the recombinant virus rHVT-VP2-HA with GFP-VP2 reverse insertion. The primers UL45-F/G2d-3F and VP2-R/UL45/46-R were used to identify viruses inserted in the reverse orientation, with target bands of 589 bp and 2,592 bp, respectively.

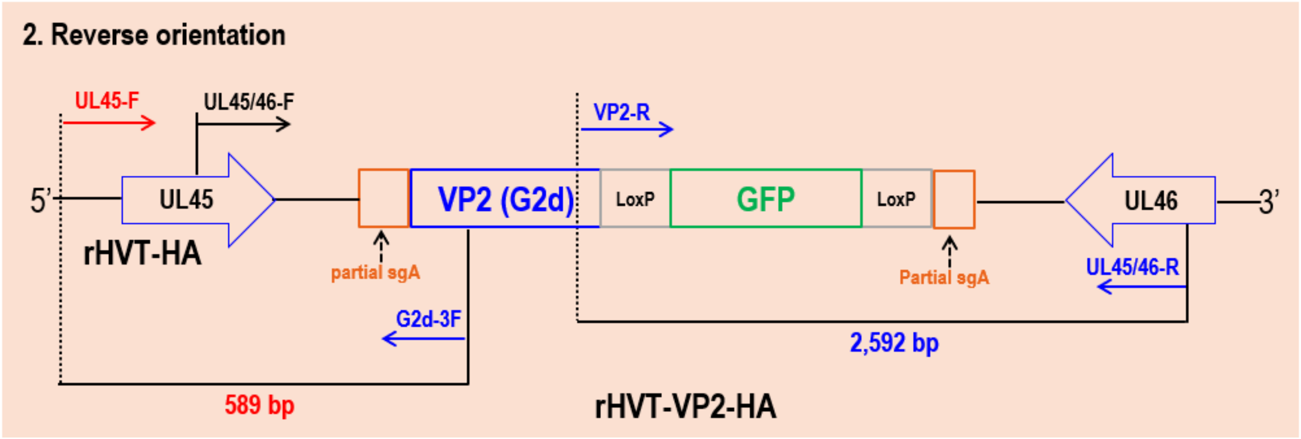

1.3 Supplementary Figure 3

Identification of GFP-VP2 insertion in rHVT-VP2-HA by PCR. (A) Identification of forward orientation (5’ junction) of rHVT-VP2-HA by PCR with the primers UL45-F/VP2-R. (B) Identification of forward orientation (3’ junction) of rHVT-VP2-HA by PCR with the primers G2d-3F/ UL45/46-R. (C) Identification of reverse orientation (5’ junction) of rHVT-VP2-HA by PCR with the primers UL45-F/G2d-3F. (D) Identification of reverse orientation (3’ junction) of rHVT-VP2-HA by PCR with the primers VP2-R /UL45/46-R.

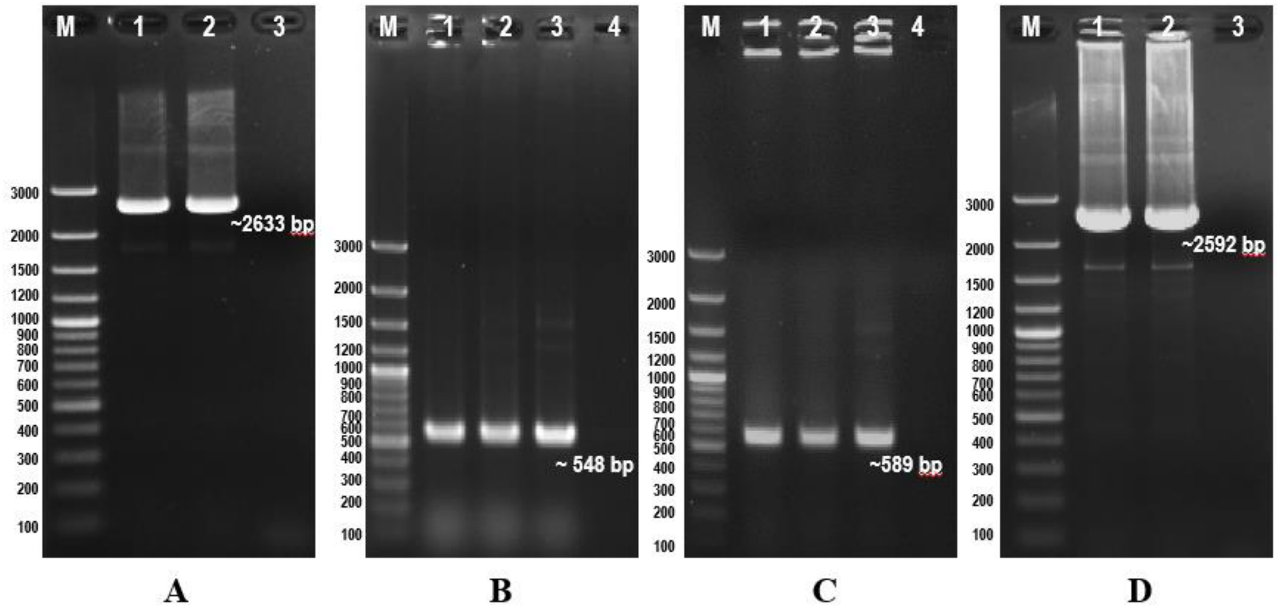

1.4 Supplementary Figure 4

Sequencing results for rHVT-VP2-HA (GFP-VP2 insertion in the forward orientation) with the primer UL45-F.

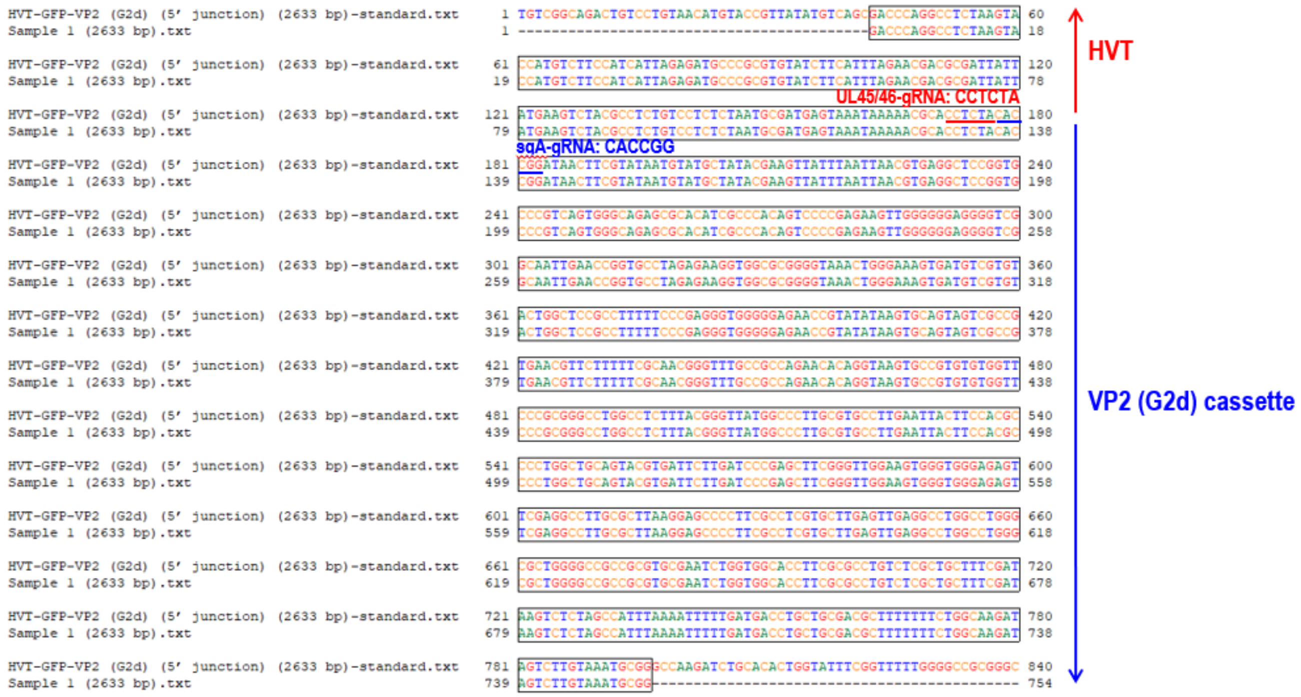

1.5 Supplementary Figure 5

Sequencing results for HVT-GFP-VP2 (GFP-VP2 insertion in the forward orientation) with the primer UL45/46-R.

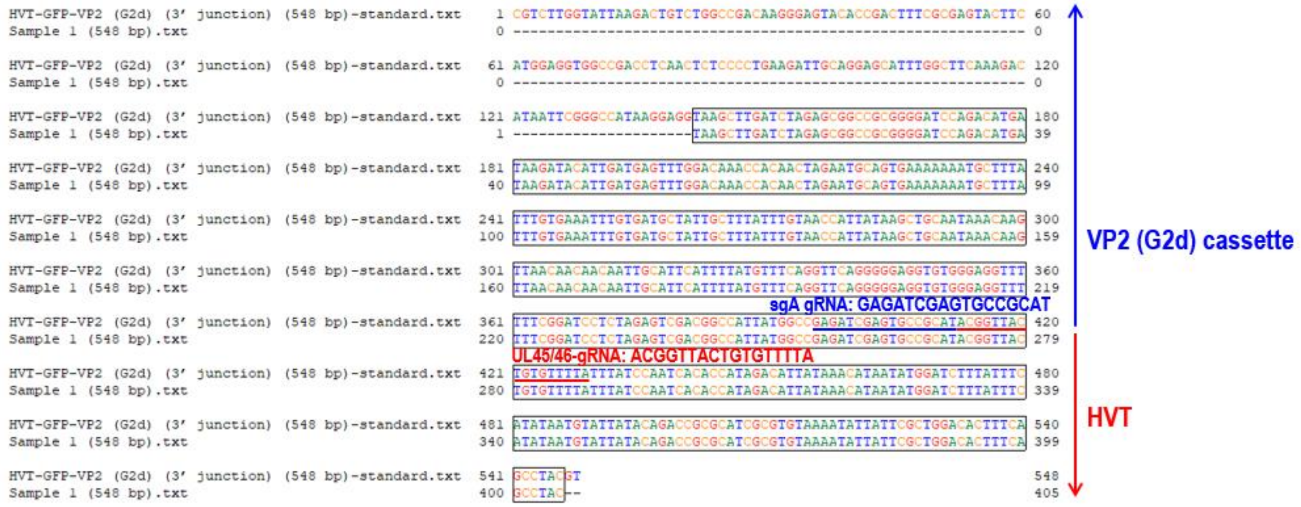

1.6 Supplementary Figure 6

Sequencing results for rHVT-VP2-HA (GFP-VP2 insertion in the reverse orientation) with the primer UL45-F.

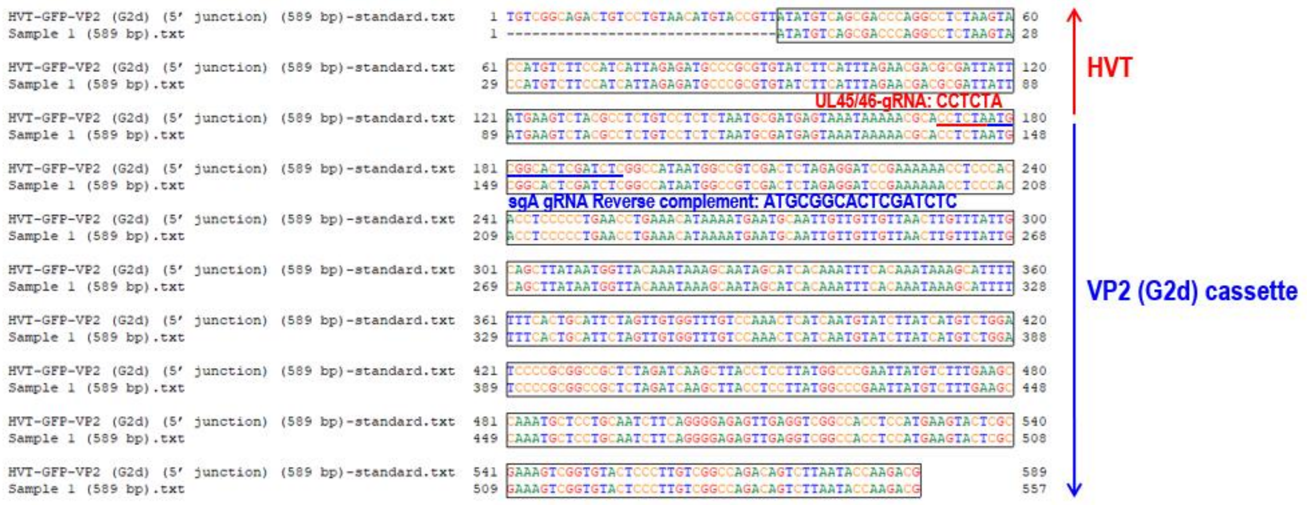

1.7 Supplementary Figure 7

Sequencing results for rHVT-VP2-HA (GFP-VP2 insertion in the reverse orientation) with the primer UL45/46-R..

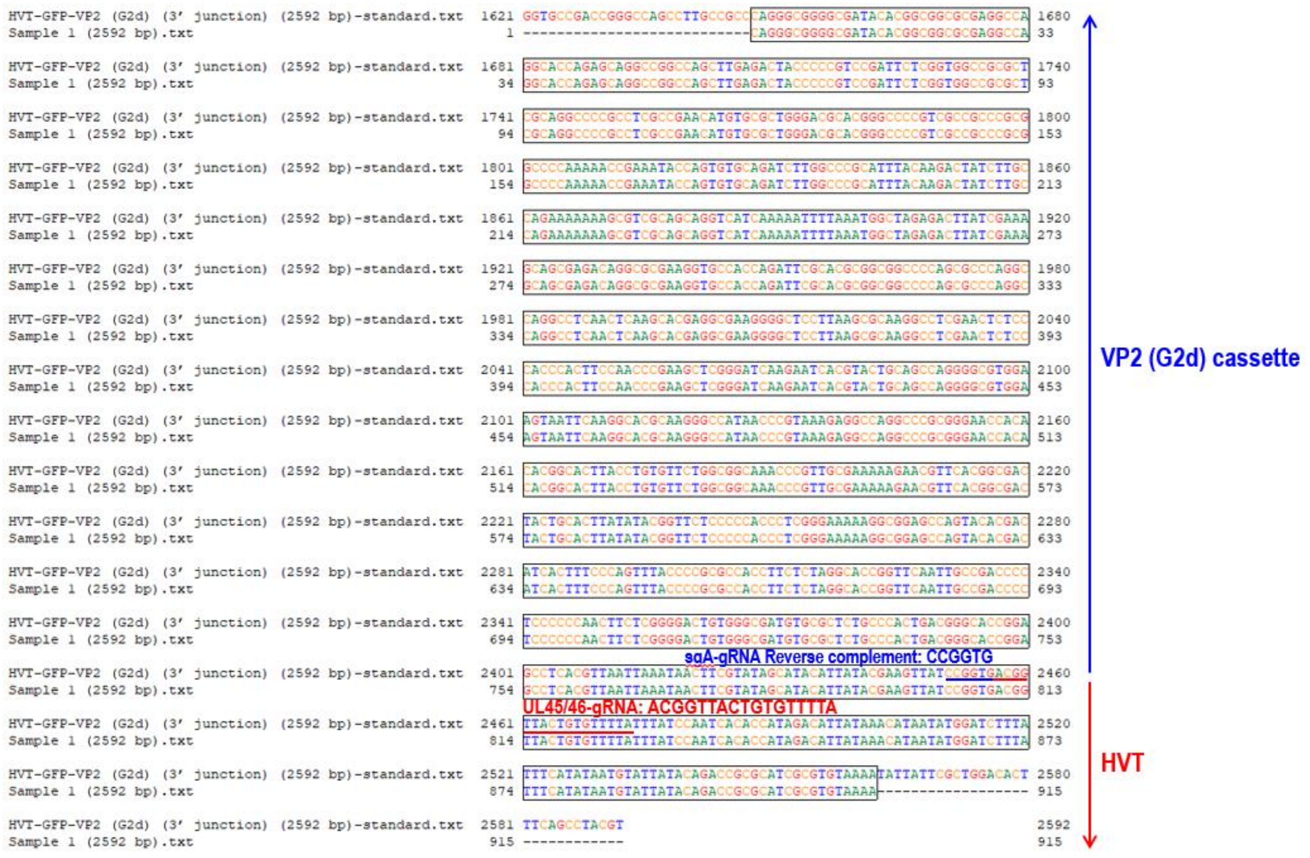

2 Supplementary Tables

2.1 Supplementary Table 1

List of primers used in this study.

Primer	Sequence (5'-3')
UL45/46-gRNA-F	CACCGAAAACACAGTAACCGTTAG
UL45/46-gRNA-R	AAACCTAACGGTTACTGTGTTTTC
sg-A-gRNA-F	CACCGAGATCGAGTGCCGCATCAC
sg-A-gRNA-R	AAACGTGATGCGGCACTCGATCTC
U6 forward	GACTATCATATGCTTACCGT
UL45-F	TGTCGGCAGACTGTCCTGTA
VP2-R	GTGCATGACCGTGCTGATTC
G2d-3F	CGTCTTGGCATCAAGACCGT
UL45/46-F	GATGCCCGCGTGTATCTTCA
UL45/46-R	ACGTAGGCTGAAAGTGTCCAG
